# Encephalopathy and brain atrophy during induction chemotherapy in acute lymphoblastic leukemia

**DOI:** 10.1002/ccr3.2969

**Published:** 2020-08-17

**Authors:** Qiao‐Ru Li, Si‐Mao Fu, Yin‐Lei Mo, Yu Li, Cong Liang, Li‐Na Wang, Wen‐Yan Tang, Li‐Bin Huang, Xue‐Qun Luo, Yan‐Lai Tang

**Affiliations:** ^1^ Department of Pediatrics Zhongshan Hospital, Sun Yat‐sen University Zhongshan China; ^2^ Department of Pediatrics The First Affiliated Hospital, Sun Yat‐sen University Guangzhou China

**Keywords:** encephalopathy, brain atrophy, chemotherapy, children, acute lymphoblastic leukemia

## Abstract

The MRI showed encephalopathy and brain atrophy of the left parietal lobe, occipital lobe and temporal lobe and decreased infiltration of the dura mater on T2‐weighted imaging. But encephalopathy and brain atrophy could be improved with neurotrophic drugs and additional intelligence teaching.

## Graphical abstract

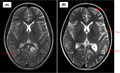

## 1 INTRODUCTION

**Figure 1:**
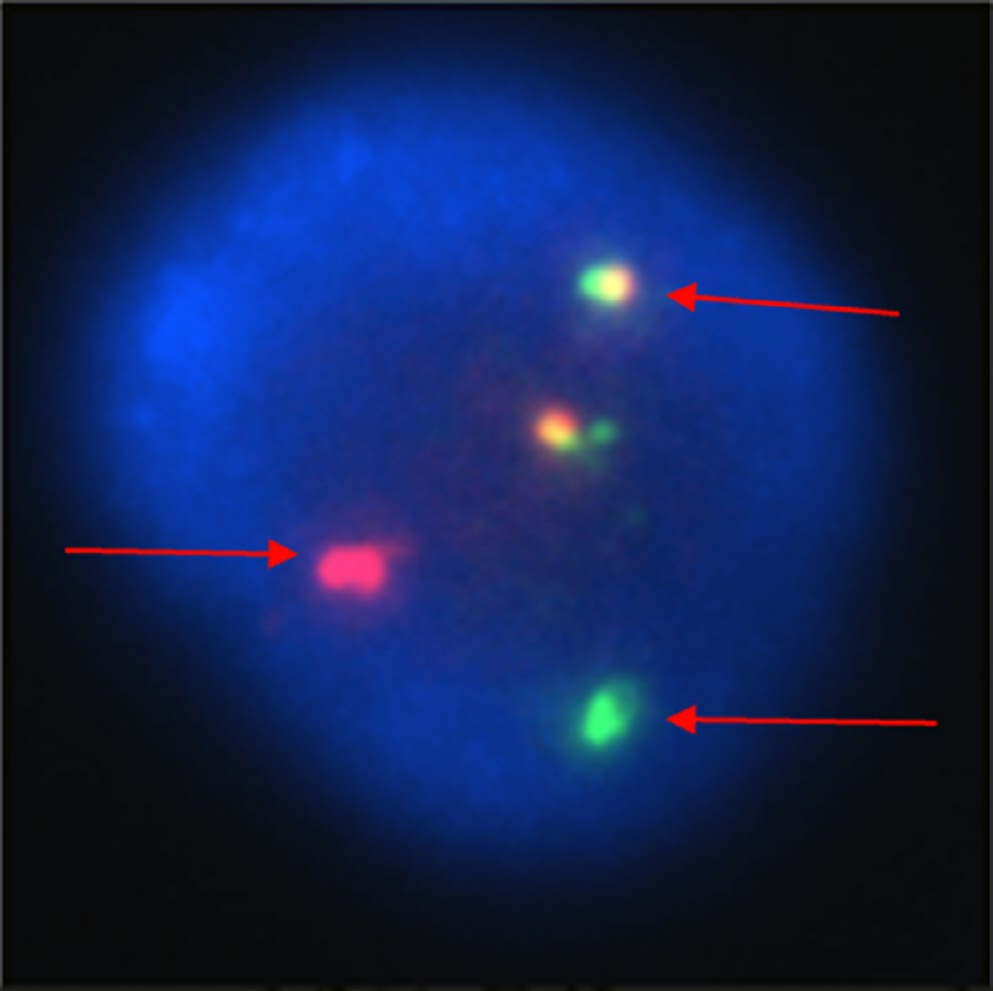
The image of a fluorescence in situ hybridization (FISH) for bone marrow examination, BCR gene was marked green, ABL1 gene was marked red, and BCR‐ABL1 fusion gene was marked yellow

**Figure 2:**
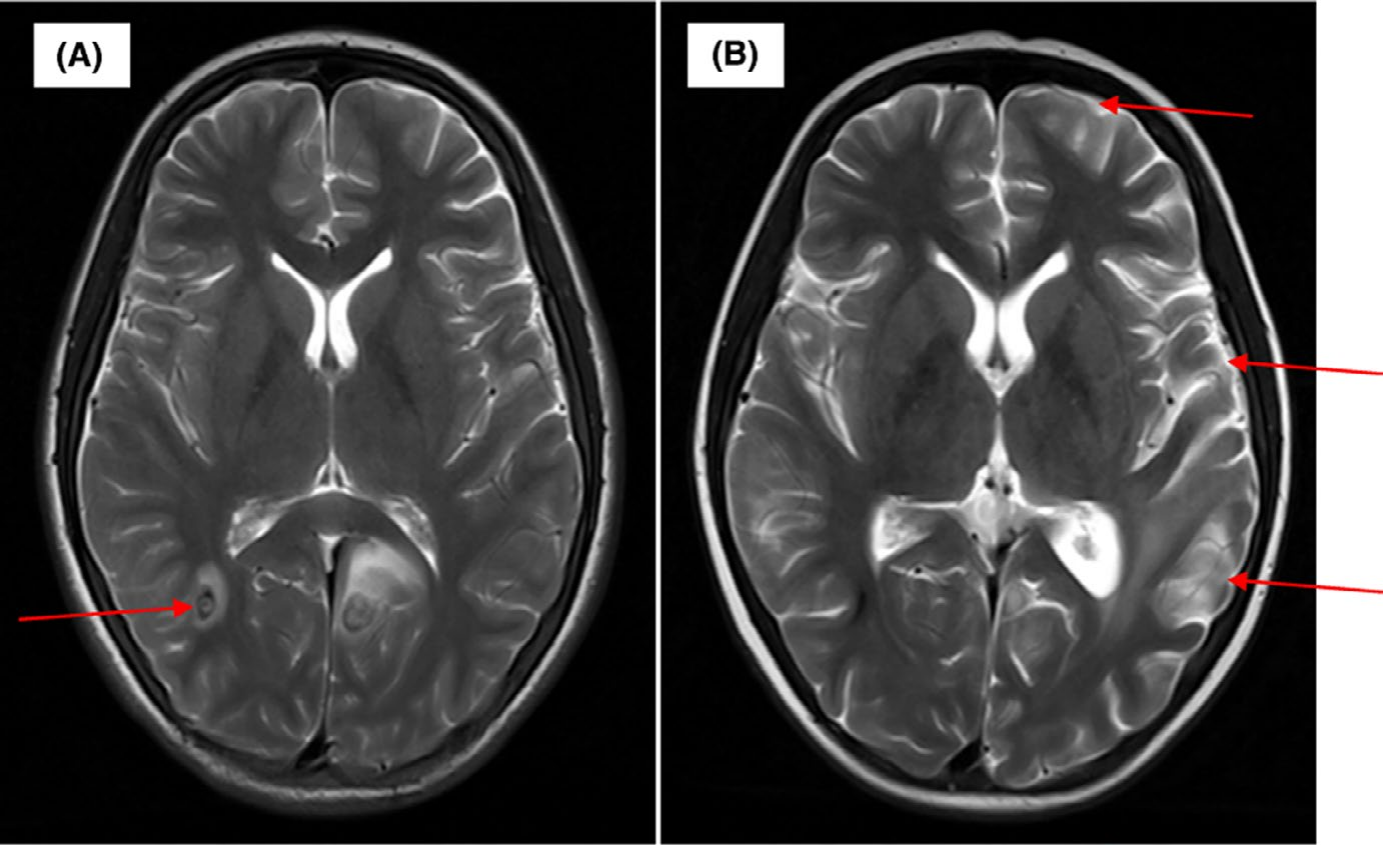
Brain MRI before chemotherapy showed extensive infiltration of the dura mater and hemorrhage of the right parietal lobe and bilateral occipital lobe on T2‐weighted imaging (A) and the MRI showed encephalopathy of the left parietal lobe, occipital lobe and temporal lobe and decreased infiltration of the dura mater on T2‐weighted imaging during chemotherapy(B)

A 14‐year‐old girl presented with irregular fever and leukocytosis (white cell count 523.96 × 10**^9^**/L). Bone marrow examination demonstrated the immune‐phenotype was common B cell acute lymphoblastic leukemia (B‐ALL). FISH showed the lymphoblast cells with positive BCR‐ABL1 fusion gene (Figure 1). The brain magnetic resonance imaging (MRI) revealed extensive infiltration of the dura mater and hemorrhage of the right parietal lobe (Figure 2A).The diagnosis of ALL was confirmed. During induction treatment with vincristine/ daunorubicin/ l‐asparaginase/dexamethasone, the patient presented with memory loss and mental decline. She was sleepy and noncommunicative. In the same time, she had encephalopathy and her intelligence deteriorated. The MRI showed encephalopathy of the left parietal lobe, occipital lobe and temporal lobe and decreased infiltration of the dura mater (Figure 2B). Fourteen days later, the memory and intelligence markedly improved with neurotrophic drugs and additional intelligence teaching. Citicoline and nerve growth factor were used for her and teach her how to live.

## CONFLICT OF INTEREST

None declared.

## AUTHOR CONTRIBUTIONS

Y‐L Tang: revised the manuscript critically. Q‐R Li, S‐M Fu, Y‐L Mo, Y Li, C Liang, L‐N Wang, W‐Y Tang, X‐Q Luo, and L‐B Huang: wrote the manuscript. All authors read and approved the final manuscript.

